# Poly[[tetra­aqua­di-μ_4_-oxalato-μ_2_-oxalato-dineo­dymium(III)] dihydrate]

**DOI:** 10.1107/S1600536812005016

**Published:** 2012-02-17

**Authors:** Gao-Juan Cao, Cheng Rong, Qing-lu Li, Wen-Jing Jiang

**Affiliations:** a College of Life Science, Fujian Agriculture and Forestry University, Fuzhou, Fujian 350002, People’s Republic of China; b Key Laboratory of Biopesticide and Chemical Biology, Ministry of Education, Fujian Agriculture and Forestry University, Fuzhou, Fujian 350002, People’s Republic of China

## Abstract

The title compound, {[Nd_2_(C_2_O_4_)_3_(H_2_O)_4_]·2H_2_O}_*n*_, was synthesized hydro­thermally in the presence of bis­(carb­oxy­ethyl­germanium) sesquioxide. It is isostructural with the corresponding Pr compound [Yang *et al.* (2009). *Acta Cryst.* E**65**, m1152–m1153]. The Nd^3+^ cation is nine-coordinated and its coordination polyhedron can be described as a distorted tricapped trigonal prism. Two Nd^3+^ ions are connected by two O atoms from two oxalate ions to give a dinuclear Nd_2_ unit. The unit is further linked to four others *via* four oxalate ions yielding a layerparallel to (0-11). The linkages between the layers by neighbouring oxalate anions lead to a three-dimensional framework with channels along the *c* axis. The coordinating and free water mol­ecules are located in the channels and make contact with each other and the host framework by weak O—H⋯O hydrogen bonds.

## Related literature

For the application of lanthanide compounds, see: Kido & Okamoto (2002 ▶). For background to lanthanide oxalates, see: Kahwa *et al.* (1984 ▶); Trombe & Jaud (2003 ▶); Wang *et al.* (2008 ▶). For the isostructural Pr compound, see: Yang *et al.* (2009 ▶).
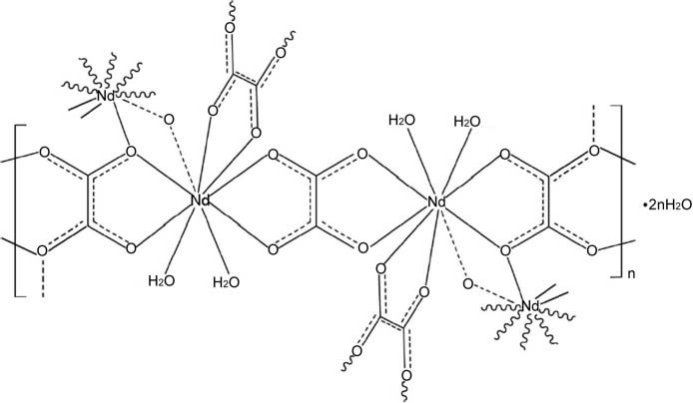


## Experimental

### 

#### Crystal data

[Nd_2_(C_2_O_4_)_3_(H_2_O)_4_]·2H_2_O
*M* *_r_* = 660.64
Triclinic, 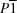

*a* = 6.036 (3) Å
*b* = 7.603 (3) Å
*c* = 8.906 (4) Å
α = 98.386 (6)°
β = 99.742 (3)°
γ = 96.802 (5)°
*V* = 394.2 (3) Å^3^
*Z* = 1
Mo *K*α radiation
μ = 6.61 mm^−1^
*T* = 293 K
0.05 × 0.05 × 0.05 mm

#### Data collection

Rigaku SCXmini diffractometer
Absorption correction: multi-scan (*SADABS*; Sheldrick, 2004 ▶) *T*_min_ = 0.758, *T*_max_ = 1.000
2997 measured reflections
1721 independent reflections
1600 reflections with *I* > 2σ(*I*)
*R*_int_ = 0.019

#### Refinement

*R*[*F* ^2^ > 2σ(*F* ^2^)] = 0.019
*wR*(*F* ^2^) = 0.045
*S* = 1.04
1721 reflections
137 parameters
9 restraints
All H-atom parameters refined
Δρ_max_ = 1.01 e Å^−3^
Δρ_min_ = −1.06 e Å^−3^

### 

Data collection: *CrystalClear* (Rigaku, 2007 ▶); cell refinement: *CrystalClear*; data reduction: *CrystalClear*; program(s) used to solve structure: *SHELXS97* (Sheldrick, 2008 ▶); program(s) used to refine structure: *SHELXL97* (Sheldrick, 2008 ▶); molecular graphics: *SHELXTL* (Sheldrick, 2008 ▶); software used to prepare material for publication: *SHELXTL*.

## Supplementary Material

Crystal structure: contains datablock(s) I, global. DOI: 10.1107/S1600536812005016/fi2122sup1.cif

Structure factors: contains datablock(s) I. DOI: 10.1107/S1600536812005016/fi2122Isup2.hkl

Additional supplementary materials:  crystallographic information; 3D view; checkCIF report

## Figures and Tables

**Table 1:** Hydrogen-bond geometry (Å, °)

*D*—H⋯*A*	*D*—H	H⋯*A*	*D*⋯*A*	*D*—H⋯*A*
O1*W*—H1⋯O5^i^	0.84 (2)	2.00 (3)	2.681 (4)	138 (4)
O1*W*—H1⋯O3^i^	0.84 (2)	2.55 (3)	3.244 (4)	141 (4)
O1*W*—H2⋯O3*W*	0.82 (2)	2.01 (2)	2.833 (4)	175 (5)
O2*W*—H3⋯O3^ii^	0.83 (2)	2.20 (3)	2.919 (4)	145 (4)
O2*W*—H4⋯O3*W*^ii^	0.83 (2)	2.00 (2)	2.809 (4)	165 (4)
O3*W*—H5⋯O4^iii^	0.82 (2)	2.04 (2)	2.827 (4)	159 (5)
O3*W*—H6⋯O6^iv^	0.85 (2)	2.10 (4)	2.835 (4)	146 (5)

Symmetry codes: (i) 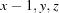; (ii) 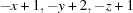; (iii) 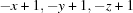; (iv) 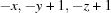.

**Fig. 1.**
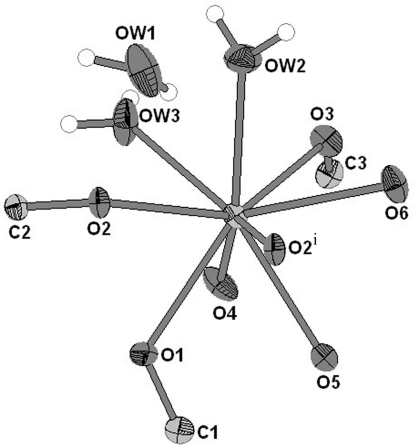
The asymmetric unit of the title complex, together with additional O2 atom to complete the coordination of the Nd3+ cation. Symmetry operation (i) 1–x, 2 - y, 2–z. Displacement ellipsoids are plotted at the 50% probability level. H atoms are shown as small circles of arbitrary radii.

**Fig. 2.**
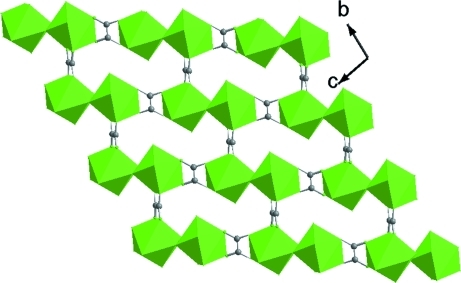
The three-dimensional framework of the title complex with channels viewed along the a axis.

**Fig. 3.**
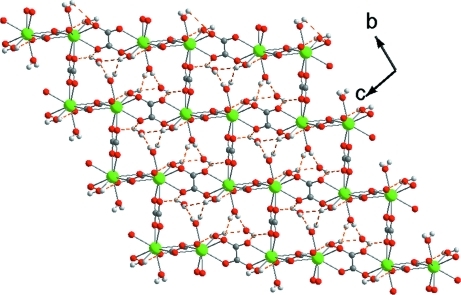
Hydrogen bonds with dashed lines in the title compound viewed along the a axis.

## supplementary crystallographic information

### Comment

The current interest in designing and making lanthanide compounds has been excitated not only by their impressive structural but also by their application in optoelectronic field (Kido & Okamoto, 2002). In our work on the preparation of lanthanide-organogermanate compounds, an unexpected lanthanide oxalate {[Nd(C_2_O_4_)_1.5_(H_2_O)_2_]^.^H_2_O}_n_, was obtained. Although the synthesis methods are different, it is isostructural with the corresponding Pr compound (Yang *et al.*, 2009).

Crystal structure determination by X-ray diffraction was performed on a SCXmini-CCD diffractometer equipped with a graphite-monochromatic Mo*Kα* (*λ* = 0.71073 Å) radiation using an *ω* scan mode at 273 K. The crystallographic analysis reveals that the asymmetric unit of this compound contains one independent Nd atom, six O atoms, three C atoms and three water molecules. The Nd^3+^ cation is nine-coordinated and is described as distorted tricapped trigonal prism: six O atoms from three C_2_O_4_^2-^ ligands, one O atom from one C_2_O_4_^2-^ and two water molecules. Two Nd^3+^ ions are connected by two O atoms from two oxalates to give a dinuclear Nd_2_ unit. The unit is further connected four others *via* four oxalates to form a layer. The linkages between the layers by oxalates lead to a three-dimensional framework with channels along *a* axis, as shown in Fig.2. The coordinated and free water molecules is located in the channels and contact with each other and host framework by O—H···O hydrogne bonds (Fig.3.).

### Experimental

A mixture of H_2_E_2_Ge_2_O_3_ (bis(carboxyethylgermanium) sesquioxide) (0.085 g), Nd_2_O_3_ (0.090 g), H_2_C_2_O_4_ (0.090 g) and H_2_O (10 ml) was stirred for about 30 min, then sealed in a 25 ml Teflonlined bomb at 170°C for 7 days, cooled to room temperature. Pink prismatic crystals of the title compound were obtained by filtration (yield about 55% based on H_2_C_2_O_4_), washed with distilled water, and dried in air. The H_2_E_2_Ge_2_O_3_ ligand plays a key role in the formation of the title compound although it is not present in the final product. If H_2_E_2_Ge_2_O_3_ ligand was not added, we obtained a different neodymium oxalate reported reviously (Trombe & Jaud, 2003). Elemental analysis calcd (%) for C_6_H_12_Nd_2_O_18_: C, 10.91; H, 1.83; Found: C, 10.87; H, 1.88.

### Refinement

All non-hydrogen atoms were refined anisotropically. The water H atoms were located in a difference Fourier map and refined with a distance restraint of O—H = 0.85 Å, and H–H=1.32Å within the water molecules and with U*_iso_*(H) = 1.5*U*_eq_(O).

### Crystal data

**Table:**

[Nd_2_(C_2_O_4_)_3_(H_2_O)_4_]·2H_2_O	*Z* = 1
*M**_r_* = 660.64	*F*(000) = 312
Triclinic, *P*1	*D*_x_ = 2.783 Mg m^−^^3^
Hall symbol: -P 1	Mo *K*α radiation, λ = 0.71073 Å
*a* = 6.036 (3) Å	Cell parameters from 1101 reflections
*b* = 7.603 (3) Å	θ = 2.4–27.5°
*c* = 8.906 (4) Å	µ = 6.61 mm^−^^1^
α = 98.386 (6)°	*T* = 293 K
β = 99.742 (3)°	Prism, pink
γ = 96.802 (5)°	0.05 × 0.05 × 0.05 mm
*V* = 394.2 (3) Å^3^	

### Data collection

**Table:**

Rigaku SCXmini diffractometer	1721 independent reflections
Radiation source: fine-focus sealed tube	1600 reflections with *I* > 2σ(*I*)
Graphite monochromator	*R*_int_ = 0.019
ω scans	θ_max_ = 27.5°, θ_min_ = 3.3°
Absorption correction: multi-scan (*SADABS*; Sheldrick, 2004)	*h* = −7→7
*T*_min_ = 0.758, *T*_max_ = 1.000	*k* = −9→9
2997 measured reflections	*l* = −11→11

### Refinement

**Table:**

Refinement on *F*^2^	Secondary atom site location: difference Fourier map
Least-squares matrix: full	Hydrogen site location: inferred from neighbouring sites
*R*[*F*^2^ > 2σ(*F*^2^)] = 0.019	All H-atom parameters refined
*wR*(*F*^2^) = 0.045	*w* = 1/[σ^2^(*F*_o_^2^) + (0.0267*P*)^2^] where *P* = (*F*_o_^2^ + 2*F*_c_^2^)/3
*S* = 1.04	(Δ/σ)_max_ = 0.001
1721 reflections	Δρ_max_ = 1.01 e Å^−^^3^
137 parameters	Δρ_min_ = −1.06 e Å^−^^3^
9 restraints	Extinction correction: *SHELXL97* (Sheldrick, 2008), Fc^*^=kFc[1+0.001xFc^2^λ^3^/sin(2θ)]^-1/4^
Primary atom site location: structure-invariant direct methods	Extinction coefficient: 0.0250 (12)

### Special details

**Table:**

Geometry. All e.s.d.'s (except the e.s.d. in the dihedral angle between two l.s. planes) are estimated using the full covariance matrix. The cell e.s.d.'s are taken into account individually in the estimation of e.s.d.'s in distances, angles and torsion angles; correlations between e.s.d.'s in cell parameters are only used when they are defined by crystal symmetry. An approximate (isotropic) treatment of cell e.s.d.'s is used for estimating e.s.d.'s involving l.s. planes.
Refinement. Refinement of *F*^2^ against ALL reflections. The weighted *R*-factor *wR* and goodness of fit *S* are based on *F*^2^, conventional *R*-factors *R* are based on *F*, with *F* set to zero for negative *F*^2^. The threshold expression of *F*^2^ > σ(*F*^2^) is used only for calculating *R*-factors(gt) *etc*. and is not relevant to the choice of reflections for refinement. *R*-factors based on *F*^2^ are statistically about twice as large as those based on *F*, and *R*- factors based on ALL data will be even larger.

### Fractional atomic coordinates and isotropic or equivalent isotropic displacement parameters (Å^2^)

**Table:**

	*x*	*y*	*z*	*U*_iso_*/*U*_eq_	
Nd	0.50918 (3)	0.80732 (2)	0.795455 (19)	0.01243 (9)	
O1	0.3007 (4)	0.6414 (3)	0.9553 (3)	0.0197 (5)	
O2	0.2687 (4)	1.0032 (3)	0.9482 (3)	0.0159 (5)	
O3	0.6296 (4)	0.7244 (3)	0.5432 (3)	0.0194 (5)	
O4	0.7188 (4)	0.5888 (3)	0.9155 (3)	0.0218 (5)	
O5	0.9212 (4)	0.8988 (3)	0.8085 (3)	0.0210 (5)	
O6	0.3574 (5)	0.4917 (3)	0.6531 (3)	0.0227 (5)	
C1	0.3797 (6)	0.5159 (4)	1.0114 (4)	0.0160 (7)	
C2	0.0540 (5)	0.9720 (4)	0.9295 (4)	0.0138 (6)	
C3	0.5789 (6)	0.5661 (4)	0.4686 (4)	0.0163 (7)	
O1W	0.1774 (4)	0.8272 (4)	0.5951 (3)	0.0303 (7)	
H1	0.052 (5)	0.846 (6)	0.618 (5)	0.045*	
H2	0.145 (7)	0.765 (6)	0.509 (3)	0.045*	
O2W	0.5617 (5)	1.0983 (4)	0.7077 (3)	0.0283 (6)	
H3	0.461 (5)	1.144 (6)	0.658 (5)	0.042*	
H4	0.680 (4)	1.171 (5)	0.722 (6)	0.042*	
O3W	0.0780 (5)	0.6307 (4)	0.2901 (4)	0.0390 (8)	
H5	0.105 (8)	0.550 (6)	0.226 (5)	0.059*	
H6	−0.065 (3)	0.620 (7)	0.277 (6)	0.059*	

### Atomic displacement parameters (Å^2^)

**Table:**

	*U*^11^	*U*^22^	*U*^33^	*U*^12^	*U*^13^	*U*^23^
Nd	0.01250 (11)	0.01253 (11)	0.01129 (11)	−0.00015 (6)	0.00267 (6)	−0.00003 (6)
O1	0.0213 (13)	0.0226 (13)	0.0199 (12)	0.0078 (10)	0.0081 (10)	0.0099 (10)
O2	0.0100 (11)	0.0192 (12)	0.0172 (11)	0.0004 (9)	0.0032 (9)	−0.0006 (9)
O3	0.0231 (13)	0.0158 (12)	0.0170 (12)	−0.0022 (10)	0.0058 (10)	−0.0027 (10)
O4	0.0176 (13)	0.0249 (13)	0.0272 (13)	0.0047 (10)	0.0086 (10)	0.0123 (11)
O5	0.0137 (11)	0.0311 (14)	0.0140 (11)	−0.0038 (10)	0.0023 (9)	−0.0034 (10)
O6	0.0303 (14)	0.0191 (12)	0.0172 (12)	−0.0045 (10)	0.0128 (10)	−0.0047 (10)
C1	0.0177 (17)	0.0169 (16)	0.0125 (15)	0.0023 (13)	0.0028 (13)	−0.0001 (13)
C2	0.0131 (15)	0.0123 (14)	0.0153 (16)	0.0008 (12)	0.0025 (12)	0.0014 (13)
C3	0.0155 (16)	0.0179 (17)	0.0135 (15)	0.0021 (13)	0.0000 (12)	0.0000 (13)
O1W	0.0161 (13)	0.0518 (19)	0.0197 (13)	0.0077 (12)	0.0025 (10)	−0.0053 (13)
O2W	0.0261 (15)	0.0227 (14)	0.0342 (16)	−0.0029 (11)	−0.0005 (12)	0.0112 (12)
O3W	0.0284 (16)	0.0440 (18)	0.0356 (17)	−0.0137 (14)	0.0118 (13)	−0.0141 (14)

### Geometric parameters (Å, º)

**Table:**

Nd—O1	2.441 (3)	O5—C2^iii^	1.241 (4)
Nd—O2W	2.455 (3)	O6—C3^iv^	1.248 (4)
Nd—O4	2.462 (3)	C1—O4^ii^	1.258 (5)
Nd—O5	2.480 (3)	C1—C1^ii^	1.542 (7)
Nd—O1W	2.481 (3)	C2—O5^v^	1.241 (4)
Nd—O3	2.494 (3)	C2—C2^vi^	1.539 (6)
Nd—O6	2.530 (3)	C3—O6^iv^	1.248 (4)
Nd—O2^i^	2.576 (2)	C3—C3^iv^	1.532 (7)
Nd—O2	2.601 (2)	O1W—H1	0.839 (19)
O1—C1	1.246 (4)	O1W—H2	0.823 (19)
O2—C2	1.267 (4)	O2W—H3	0.830 (18)
O2—Nd^i^	2.576 (2)	O2W—H4	0.827 (19)
O3—C3	1.263 (4)	O3W—H5	0.824 (19)
O4—C1^ii^	1.258 (5)	O3W—H6	0.845 (19)
			
O1—Nd—O2W	142.79 (9)	O4—Nd—O2	120.99 (8)
O1—Nd—O4	66.13 (8)	O5—Nd—O2	122.26 (8)
O2W—Nd—O4	142.62 (9)	O1W—Nd—O2	76.88 (8)
O1—Nd—O5	132.18 (8)	O3—Nd—O2	146.24 (8)
O2W—Nd—O5	71.58 (9)	O6—Nd—O2	122.82 (8)
O4—Nd—O5	71.43 (9)	O2^i^—Nd—O2	65.42 (9)
O1—Nd—O1W	97.08 (10)	C1—O1—Nd	120.1 (2)
O2W—Nd—O1W	70.46 (10)	C2—O2—Nd^i^	118.3 (2)
O4—Nd—O1W	142.10 (10)	C2—O2—Nd	123.74 (19)
O5—Nd—O1W	130.44 (10)	Nd^i^—O2—Nd	114.58 (8)
O1—Nd—O3	134.13 (8)	C3—O3—Nd	121.0 (2)
O2W—Nd—O3	77.81 (9)	C1^ii^—O4—Nd	119.4 (2)
O4—Nd—O3	92.73 (9)	C2^iii^—O5—Nd	123.2 (2)
O5—Nd—O3	67.13 (8)	C3^iv^—O6—Nd	120.3 (2)
O1W—Nd—O3	74.66 (9)	O1—C1—O4^ii^	126.2 (3)
O1—Nd—O6	70.15 (8)	O1—C1—C1^ii^	117.3 (4)
O2W—Nd—O6	132.48 (9)	O4^ii^—C1—C1^ii^	116.5 (4)
O4—Nd—O6	69.77 (9)	O5^v^—C2—O2	126.3 (3)
O5—Nd—O6	114.40 (8)	O5^v^—C2—C2^vi^	116.4 (4)
O1W—Nd—O6	72.59 (10)	O2—C2—C2^vi^	117.2 (3)
O3—Nd—O6	64.28 (8)	O6^iv^—C3—O3	125.9 (3)
O1—Nd—O2^i^	85.95 (9)	O6^iv^—C3—C3^iv^	117.0 (4)
O2W—Nd—O2^i^	81.77 (9)	O3—C3—C3^iv^	117.1 (4)
O4—Nd—O2^i^	77.35 (8)	Nd—O1W—H1	122 (3)
O5—Nd—O2^i^	63.77 (7)	Nd—O1W—H2	124 (3)
O1W—Nd—O2^i^	137.60 (8)	H1—O1W—H2	105 (3)
O3—Nd—O2^i^	130.55 (8)	Nd—O2W—H3	126 (3)
O6—Nd—O2^i^	144.97 (8)	Nd—O2W—H4	128 (3)
O1—Nd—O2	67.11 (8)	H3—O2W—H4	106 (3)
O2W—Nd—O2	75.84 (10)	H5—O3W—H6	105 (3)

Symmetry codes: (i) −*x*+1, −*y*+2, −*z*+2; (ii) −*x*+1, −*y*+1, −*z*+2; (iii) *x*+1, *y*, *z*; (iv) −*x*+1, −*y*+1, −*z*+1; (v) *x*−1, *y*, *z*; (vi) −*x*, −*y*+2, −*z*+2.

### Hydrogen-bond geometry (Å, º)

**Table:**

*D*—H···*A*	*D*—H	H···*A*	*D*···*A*	*D*—H···*A*
O1*W*—H1···O5^v^	0.84 (2)	2.00 (3)	2.681 (4)	138 (4)
O1*W*—H1···O3^v^	0.84 (2)	2.55 (3)	3.244 (4)	141 (4)
O1*W*—H2···O3*W*	0.82 (2)	2.01 (2)	2.833 (4)	175 (5)
O2*W*—H3···O3^vii^	0.83 (2)	2.20 (3)	2.919 (4)	145 (4)
O2*W*—H4···O3*W*^vii^	0.83 (2)	2.00 (2)	2.809 (4)	165 (4)
O3*W*—H5···O4^iv^	0.82 (2)	2.04 (2)	2.827 (4)	159 (5)
O3*W*—H6···O6^viii^	0.85 (2)	2.10 (4)	2.835 (4)	146 (5)

Symmetry codes: (iv) −*x*+1, −*y*+1, −*z*+1; (v) *x*−1, *y*, *z*; (vii) −*x*+1, −*y*+2, −*z*+1; (viii) −*x*, −*y*+1, −*z*+1.
